# A Culture of Early Mobilization in Adult Intensive Care Units: Perspective and Competency of Physicians

**DOI:** 10.3390/healthcare12131300

**Published:** 2024-06-28

**Authors:** Ali Albarrati, Monira I. Aldhahi, Turki Almuhaid, Ali Alnahdi, Ahmed S. Alanazi, Abdulfattah S. Alqahtani, Rakan I. Nazer

**Affiliations:** 1 Rehabilitation Sciences Department, College of Applied Medical Sciences, King Saud University, P.O. Box 10219, Riyadh 11433, Saudi Arabia; alialnahdi@ksu.edu.sa (A.A.); abalqahtani@ksu.edu.sa (A.S.A.); 2 Department of Rehabilitation Sciences, College of Health and Rehabilitation Sciences, Princess Nourah bint Abdulrahman University, P.O. Box 84428, Riyadh 11671, Saudi Arabia; mialdhahi@pnu.edu.sa; 3 Prince Mohammed Bin Abdulaziz Hospital, Ministry of Health, Riyadh 14214, Saudi Arabia; almuhaidt@pmah.med.sa; 4 Department of Physical Therapy, College of Medical Rehabilitation Sciences, Taibah University, Medina 42353, Saudi Arabia; asanazi@taibahu.edu.sa; 5 Cardiac Sciences Department, College of Medicine, King Saud University, Riyadh 11421, Saudi Arabia; raknazer@ksu.edu.sa

**Keywords:** physician’s knowledge, adult intensive care unit, early mobilization, health facilities, ambulation, patient care

## Abstract

Background: Early mobility (EM) is vital in the intensive care unit (ICU) to counteract immobility-related effects. A multidisciplinary approach is key, as it requires precise initiation knowledge. However, physicians’ understanding of EM in adult ICU settings remains unexplored. This study was conducted to investigate the knowledge and clinical competency of physicians working in adult ICUs toward EM. Methods: This cross-sectional study enrolled 236 physicians to assess their knowledge of EM. A rigorously designed survey comprising 30 questions across the demographic, theoretical, and clinical domains was employed. The criteria for knowledge and competency were aligned with the minimum passing score (70%) stipulated for physician licensure by the medical regulatory authority in Saudi Arabia. Results: Nearly 40% of the respondents had more than 5 years of experience. One-third of the respondents received theoretical knowledge about EM as part of their residency training, and only 4% of the respondents attended formal courses to enhance their knowledge. Almost all the respondents (95%) stated their awareness of EM benefits and its indications and contraindications and considered it safe to mobilize patients on mechanical ventilators. However, 62.3% of the respondents did not support EM for critically ill patients on mechanical ventilators until weaning. In contrast, 51.7% of respondents advised EM for agitated patients with RASS > 2. Only 113 (47.9%) physicians were competent in determining the suitability of ICU patients for EM. For critically ill patients who should be mobilized, nearly 60% of physicians refused to initiate EM. Conclusions: This study underscores insufficient practical knowledge of ICU physicians about EM criteria, which leads to suboptimal decisions, particularly in complex ICU cases. These findings emphasize the need for enhanced training and education of physicians working in adult ICU settings to optimize patient care and outcomes in critical care settings.

## 1. Introduction

Prolonged immobility or bed rest has many detrimental effects on the patient’s health. Brower (2009) reported that progressive immobility could result in muscular atrophy and advanced deconditioning [1]. Early mobilization (EM) of critically ill patients is a crucial aspect of intensive care management [2] and has been proven to enhance patient outcomes, reduce hospital stay duration, and hospital expenses [3]. EM and rehabilitation in the intensive care unit (ICU) have several positive physical, physiological, and psychological effects that prevent the complications associated with ICU immobility. These are characterized by accelerating weaning time, reducing delirium, maintaining central and peripheral musculoskeletal integrity, reducing the risk of polyneuropathy and myopathy, reducing the length of ICU stays and hospitalizations, and reducing mortality during hospitalization [4,5,6,7].

Although EM has demonstrated numerous benefits, its successful implementation requires the active participation and competency of healthcare professionals, particularly physicians, who play a crucial role in decision-making and patient care within the ICU setting. Despite the benefits of EM, healthcare providers face many challenges in initiating and incorporating EM into ICUs. Potential barriers include inadequate ICU physicians’ knowledge of EM and poor interdisciplinary communication [8]. This has led to a delay in the initiation of EM worldwide and has subsequently increased morbidity and mortality among patients in ICU settings. 

To ensure optimal patient outcomes and promote a culture of early mobilization, it is essential to assess the knowledge and competency of physicians working in adult ICUs for early mobilization. Physicians’ perspectives on early mobilization are influenced by various factors, including their training, knowledge of the evidence supporting its benefits, perceived patient safety concerns, and competing priorities in the ICU. Addressing these factors is crucial for overcoming barriers and effectively integrating early mobilization into the routine care of critically ill patients. Understanding the perspective and competency of physicians in relation to early mobilization is critical for achieving positive patient outcomes and improving the quality of care provided in adult ICUs. By identifying barriers, addressing knowledge gaps, and promoting a collaborative approach among healthcare professionals, we can establish a culture that embraces and prioritizes early mobilization as an essential component of care for critically ill patients in the ICU. Therefore, this study aims to examine the knowledge and clinical competency of physicians working in adult ICU settings toward the initiation of EM. 

## 2. Method

### 2.1. Study Design

This cross-sectional study focused on gaining knowledge of the critical aspects of EM among physicians working in ICUs by surveying their knowledge and clinical competency regarding EM in adult ICU settings. This study was approved by the Research Ethics Committee of King Saud University (IRB Number: 22/0178), and all participants provided consent prior to participation.

### 2.2. Participants and Setting

The survey was distributed via the databases of the recognized official medical societies under the umbrella of the Saudi Commission for Health Specialties (https://scfhs.org.sa/en, accessed on 1 January 2023). Participants were asked to provide their professional classification and registration electronic numbers provided by the Saudi Commission for Health Specialties. 

To be eligible for inclusion in the study, physicians had to have a minimum of six months of experience in an adult ICU setting and specialties of cardiology, pulmonology, cardiac surgery, critical care medicine, thoracic surgery, general surgery, anesthesia, and internal medicine. Physicians who were not currently practicing, had less than six months of experience, or were interns in an adult ICU setting were excluded from the study. The sample size was estimated using the formula: N = (Z_a/2_)^2^ s^2^/d^2^ [9].

Standard deviation = 21.3, acceptable error = 3, confidence level = 95%, and sample size = 194. The standard deviation was obtained from a previous study by Qutub et al. (2018) [10]. The snowball sampling method was used in this study, which required the participants to distribute it themselves as well. 

### 2.3. Development of the Survey

To assess ICU physicians’ knowledge of EM principles and practical competency, the survey was divided into three categories: demographic and clinical background, EM knowledge, and ICU clinical cases (Appendix A). The selection of the questionnaire was based on clinical practice guidelines for EM in the ICU, as well as expert consensus and recommendations, taking into account levels of difficulty and discrimination. Questions were categorized based on difficulty levels (simple, difficult, complicated) [11,12,13].

The analytical report of the question pool was scrutinized and analyzed according to difficulty categories and discrimination indices, indicating how effectively the questions were formulated and previously tested. The discrimination index, categorized as good (greater than 0.3), fair (between 0.1 and 0.3), and poor (less than 0.1), helps differentiate the knowledge levels between physicians with varying levels of expertise. Questions with discrimination values falling into the good and fair categories are known to effectively distinguish between physicians with higher and lower levels of knowledge. The survey was piloted on ten physicians serving in adult medical and surgical ICU settings who confirmed that the generated questions were clear, understandable, and valid for the purpose of the study.

### 2.4. Data Analysis

Statistical Package for Social Sciences (SPSS) version 26.0 was used in the analysis. The basic method of frequency and percentage distribution with visualization was used for the presentation of data. The Cronbach’s alpha statistic was employed to ensure that the data were reliable and internally consistent. The Cronbach’s alpha statistic ranges from 1 to 0, and a value near 1 suggests that the data are reliable; in contrast, a value near 0 suggests that the data are not reliable. A minimum score of 70% of correct answers was required to achieve “good” knowledge and clinical competency in clinical cases. A *p*-value of less than 0.05 was considered statistically significant.

## 3. Results

### 3.1. Description of the Participants 

In total, 236 participants (181 males) were included in this study. Predominantly, the respondents (97.5%) were affiliated with governmental sectors, while a minority (2.5%) worked within the private sector. The majority (76.7%) obtained advanced medical training domestically, with 23.3% pursuing their higher medical education abroad. Diverse specialties, encompassing intensivists, were represented in Saudi Arabian ICU settings (Table 1).

### 3.2. Theoretical Knowledge about Early Mobilization in the ICU

Over half of the respondents (n = 128, 54.2%) received theoretical knowledge about EM in the ICU through their undergraduate program, while one-third (n = 81, 34.3%) received it as a part of their residency program. A minority (9, 3.8%) had taken courses or workshops on EM. In this study, (n = 12, 5.1%) reported they had no knowledge about the contraindications of EM in ICU settings. Notably, a substantial 62.3% of the respondents deferred EM for critically ill patients under mechanical ventilation until after weaning. In contrast, 51.7% recommended EM for agitated patients with a Richmond Agitation-Sedation Scale (RASS) score exceeding +2. Similar patterns were observed across various facets of physicians’ comprehension of EM within adult medical and surgical ICU settings (Table 2).

### 3.3. Clinical Competency of Physicians Working in ICU about EM

The respondents had varying opinions regarding the EM of patients with different clinical scenarios. Differences among physicians increased as the difficulty of the case scenario became more complicated (Table 3).

### 3.4. Determining Theoretical and Clinical Competency of Physicians Working in Adults ICU Settings

We screened the data to determine the knowledge and clinical competency of physicians working in adult ICU settings based on a cut-off point of 70% to be knowledgeable and clinically competent in EM in adult ICU settings. Only 113 (47.9%) of physicians demonstrated basic knowledge about EM. 

The cross-sectional survey encompassed a diverse group of specialists, including anesthetists, internists, general surgeons, thoracic surgeons, cardiac surgeons, cardiologists, pulmonologists, and intensivists. It was noted that, on average, 101 out of 129 intensivists had adequate theoretical knowledge, while only 85 (36%) intensivists could correctly recognize patients who were fit for EM. Cardiac surgeons demonstrated adequate theoretical knowledge and clinical competency about EM. In contrast, half of the general surgeons had less knowledge and clinical competency regarding EM and when the patient should be mobilized. Similarly, internists had less knowledge and were less competent in advising for EM. Other specialties are presented in Table 4.

## 4. Discussion

The results of the study indicate that there is an insufficient level of clinical competency among physicians working in adult ICU settings in Saudi Arabia toward EM. Furthermore, the findings of the current study highlight the discrepancies between physicians’ clinical competencies when EM should be initiated. 

ICU rehabilitation could play a crucial role in helping reduce both the duration of mechanical ventilation and the length of ICU and hospital stays and enhance patients’ return to functional independence. The findings of this study showed that 99.2% of physicians agreed that ICU rehabilitation was a key part of ICU care. Similar results were reported by Zão [14], who stated that ICU rehabilitation was an integral part of ICU care. Initiating EM 24–48 h after stabilization of patients is essential to avoid complications associated with immobility and post ICU syndrome [15]. Physicians working in medical and surgical ICU settings must be aware of the impact of EM to ensure that they provide medical-guided care to patients individually and when practicing in a multidisciplinary team. We found in our study that 94% of physicians agreed that oxygen saturation and lung compliance increased with EM, and serious illness and prolonged mechanical ventilation were major risk factors associated with respiratory complications and prolonged immobility. 

Moreover, nearly all physicians working in adult medical and surgical ICU settings were aware of the benefits of reducing ICU length of stay and early liberation from mechanical ventilation, and EM reduced delirium and enhanced patient awareness. These findings aligned with previous literature that indicated that proper and timely mobility in the ICU reduces the ICU length of stay, time spent on mechanical ventilation, and delirium and improves patient awareness [16]. A study by Qutub et al. [10] among respiratory care professionals recognized that knowledge was more important for EM. Therefore, healthcare providers working in different ICU settings must be equipped with essential theoretical and clinical knowledge to incorporate it into their clinical decision-making. 

In the current study, 60% of the physicians mentioned that patients on mechanical ventilators and the critically ill should be mobilized only after weaning. It is important to note that the statement suggesting that patients on mechanical ventilators and the critically ill should only be mobilized after weaning is not universally valid. It is crucial to consider that recent research and clinical guidelines practice have challenged this notion. Emerging evidence has demonstrated the potential benefits of EM, even for patients on mechanical ventilators and those who are critically ill. However, caution should be exercised in specific cases, such as patients with unstable medical conditions or those requiring strict ventilator management. Ample evidence has shown that EM contributes to improved outcomes, such as reduced ventilator-associated complications, shortened ICU length of stay, enhanced central and peripheral muscle strength, and decreased risk of functional decline and disability [6,17,18,19]. Engaging patients in appropriate physical activity and mobility exercises early in their ICU stay can help mitigate the detrimental effects of prolonged bed rest and immobility. Preventing problems associated with bed rest, promoting early ventilator weaning in a timely way, increasing patients’ general strength and endurance, and reducing hospital length of stay are all positive clinical outcomes of an EM strategy in the ICU [20]. The research conducted by Taito et al. [21] demonstrated that EM of mechanically ventilated patients in ICUs was a secure, advantageous, efficient, and successful approach that led to decreased durations on mechanical ventilation and the hospital length of stay. These benefits have been observed to surpass the occurrences of unfavorable events during EM. A number of studies have reported that physicians’ practical knowledge of EM is crucial and minimizes the occurrence of adverse events and complications associated with prolonged immobility [22,23]. Recent ICU guidelines and expert consensus and recommendations emphasize that physicians working in different ICU settings should keep continuously updated about EM criteria and adhere to multidisciplinary clinical decision-making to provide optimum patient-centered care. A cross-sectional study by Akhtar and Deshmukh [23] found that limited practical knowledge about EM resulted in prolonged ICU length of stay and consequently led to both physical and physiological manifestations along with poor quality of life.

Our results demonstrated a lower level of clinical competency among physicians working in medical and surgical ICU settings toward initiation of EM, particularly in challenging and complicated cases. In the current study, we determined the physicians’ clinical competency score based on the Saudi Commission for Health Specialties (SCFHS) minimum score to be qualified as a healthcare provider. Physicians who scored more than 70% in problem-solving case studies were deemed clinically competent in EM. Out of the total participated physicians, only 113 (47.9%) demonstrated clinical competency in EM by providing correct answers in over 70% of the clinical cases. Several potential factors may contribute to the observed lower competency levels in initiating EM, such as lack of emphasis on EM training during ICU residency programs, the level of physician experience, and lack of physicians’ exposure to severely critically ill patients. A previous study by Koo et al. [24] reported that very few physicians had knowledge about EM, regardless of their discipline or clinical experience. Inadequate knowledge and absence of guiding protocols for EM necessitate standardization of proper and timely mobility in the ICU based on the current ICU evidence-based clinical guidelines. Healthcare providers need to comprehend the significance of EM and its contribution towards better ICU patient care outcomes. 

A multidisciplinary team is needed for initiating EM for patients admitted to the ICU, including physicians, ICU nurses, respiratory therapists, physical and occupational therapists, and clinical pharmacists. A physician-driven protocol is one component of this coordinated strategy to expedite the process of getting the patient out of bed. Levels of sedation must be lowered to a point where the patient is mildly sedated to expedite patient mobility. Then, spontaneous awakening trials (SATs) and spontaneous breathing trials (SBTs) should be performed. More screening is required to evaluate whether a patient is eligible for EM. In our study, half of the physicians agreed that ABCDEF bundles were a multidisciplinary team approach to EM in ICUs and include Assess, Prevent, and Manage Pain (A), Both SATs and Spontaneous Breathing Trials (SBT) (B), Choice of analgesia and sedation (C), Delirium: Assess, Prevent, and Manage (D), EM and Exercise (E), and Family engagement and empowerment (F). Our results are in line with previous studies that emphasize the significance of ABCDEF bundles [11,21,25]. 

In this cross-sectional survey, 94.9 percent of physicians showed high knowledge in understanding the indications and contraindications of EM in the ICU. However, when tested, their knowledge of clinical case scenarios in which the critical thinking skill has to play a role in making the decision, less than half of the physicians in the survey provided the correct answer. Only 43.2% (n = 113) of the physicians had appropriate responses to different clinical case scenarios, which aimed to test the physicians’ knowledge of the indications and contraindications of EM and when it should be initiated. The high number of physicians with limited clinical awareness about the applications of EM fundamentally emphasizes the need to build clinical strategies within ICU settings to enhance the clinical practice of EM. We found in this study that cardiac surgeons demonstrated the highest level of accuracy in both theoretical and clinical knowledge. Their proficiency can be attributed to their extensive experience in managing critically ill patients, enabling them to make well-informed decisions regarding the optimal timing for implementing EM interventions, thereby enhancing patient outcomes. Other specialists who demonstrated a notable level of accuracy in initiating appropriate EM decisions included cardiologists, pulmonologists, and thoracic surgeons. These specialists were more likely to be involved in multidisciplinary teams responsible for the comprehensive care of critically ill patients, thereby gaining valuable exposure to the necessary protocols. Conversely, half of the general surgeons revealed a need for greater awareness regarding the clinical application of EM, suggesting a limited familiarity with ICU management protocols. Consequently, the long-term care of critically ill patients is often delegated to other specialists, such as intensivists.

The responsibility for the care of critically ill patients ultimately lies with intensivists, who are entrusted with making critical decisions. Therefore, it is imperative that intensivists possess a comprehensive understanding of the various protocols involved in ICU management. However, our findings revealed that one-third of intensivists required an increased awareness regarding the appropriate clinical application of EM. This finding raises some concerns, as intensivists play a pivotal role in EM decision-making. This fact highlights an important area for improvement in some ICU practices that some intensivists still need to fully grasp EM protocols and ultimately increase the overall quality of care for patients admitted to the ICU.

To enhance the EM of ICU patients among physicians, it is suggested to offer comprehensive training programs and workshops specifically focused on the significance of EM in the ICU setting. Comprehensive training should include practical demonstrations, case studies, and evidence-based guidelines for implementing EM protocols. Encourage physicians to engage in continuous professional development activities related to critical care and early mobilization. This can include attending relevant conferences, participating in webinars, and staying updated with the latest research and best practices in the field. It is required to enhance collaboration between physicians, physical therapists, and other healthcare professionals involved in patient care. Foster a multidisciplinary approach to ensure effective communication and coordination in implementing EM plans. Implementing a system for regular performance assessments of physicians regarding EM is required. This can include periodic evaluations of their understanding and clinical competency in initiating and managing EM interventions. Feedback and support should be provided to physicians to address any identified areas of improvement. By implementing these recommendations, it is possible to enhance physicians’ competency and knowledge regarding EM in the ICU, ultimately leading to improved patient outcomes and enhanced overall care within the critical care setting.

We would like to acknowledge the limitation of this study, which includes the inability to establish causality due to the cross-sectional nature of the data. The cross-sectional design of the study does not establish causality and underlying the lack of clinical competency among physicians and makes it hard to draw definitive conclusions about the cause-and-effect relationship between knowledge/attitudes and EM practices. The study sample may not fully represent the entire population of physicians involved in adult ICU settings in Saudi Arabia. It is crucial to consider these limitations when interpreting the findings of our study, and further research utilizing longitudinal or experimental designs may be warranted to address these limitations in future studies.

## 5. Conclusions

This study shows that physicians working in adult ICU settings have adequate theoretical knowledge of EM, except for general surgeons who seldom visit the ICU. The decision to initiate EM for adult patients in medical and surgical ICU settings varies among physicians, especially intensivists and general surgeons who were against EM for certain suitable ICU cases. Establishing an ICU multidisciplinary team that makes a shared clinical decision for patients in medical and surgical ICU settings based on clinical guidelines for EM in the ICU is mandatory.

## Figures and Tables

**Table 1:** Description of the participants.

Variables	Frequency (n)	Percentage (%)
**Country of the Highest Degree**		
Saudi Arabia	181	76.7
Other	55	23.3
Total	236	100.0
**Professional Status**		
Senior Resident	139	58.9
Specialist	71	30.1
Consultant	26	11.0
Total	236	100.0
**Specialty**		
Intensivist	129	54.7
Pulmonologist	2	0.8
Cardiologist	12	5.1
Cardiac surgeon	5	2.1
Thoracic surgeon	7	3.0
Anesthetist	14	5.9
Internist	53	22.5
General surgeon	14	5.9
**Experience (years)**		
Less than 5	143	60.6
5–10	22	9.3
10–15	50	21.2
15–20	18	7.6
More than 20	3	1.3

**Table 2:** Knowledge of physicians regarding early mobilization in adult ICU settings in Saudi Arabia.

Knowledge	Provided Correct Answer	Failed	Do Not Know
	n (%)	n (%)	n (%)
Know (contra)indications of EM in the ICU	224 (94.9)	5 (2.1)	7(3.0)
Start EM 24–48 h after ICU admission	225 (95.3)	6 (2.5)	5 (2.1)
Start EM only if patients are weaned from ventilators	89 (37.7)	138 (58.5)	9 (3.8)
ICU rehabilitation is a key part of ICU care	234 (99.2)	1 (0.4)	1 (0.4)
EM increases oxygen saturation/lung compliance	221 (93.6)	6 (2.5)	9 (3.8)
EM reduces ICU length of stay and time on ventilator	228 (96.6)	3 (1.3)	5 (2.1)
EM reduces postoperative delirium	224 (94.9)	3 (1.3)	9 (3.8)
It is safe and viable to mobilize patients on mechanical ventilators	209 (88.6)	18 (7.6)	9 (3.8)
It is safe to mobilize agitated patients (RASS more than +2)	96 (40.7)	122 (51.7)	18 (7.6)
The ABCDEF bundle is a multidisciplinary team approach	120 (50.8)	17 (7.2)	99 (41.9)

**Table 3:** Clinical competency of physicians regarding early mobilization of patients.

Is the Patient a Candidate for Early Mobilization	Provided Correct Answer	Failed	Do Not Know
	n (%)	n (%)	n (%)
Simple Cases			
Case-1	225 (95.3)	2 (0.8)	9 (3.8)
Case-2	197 (83.5)	27 (11.4)	12 (5.1)
Case-3	199 (84.3)	23 (9.7)	14 (5.9)
Case-4	189 (80.1)	33 (14)	14 (5.9)
Difficult Cases			
Case-5	100 (42.4)	111 (47)	25 (10.6)
Case-6	208 (88.1)	16 (6.8)	12 (5.1)
Challenging Cases			
Case-7	97 (41.1)	103 (43.6)	36 (15.3)
Case-8	201 (85.2)	25 (10.6)	10 (4.2)
Complicated Cases			
Case-9	80 (33.9)	142 (60.2)	14 (5.9)
Case-10	99 (41.9)	86 (36.4)	51 (21.6)

**Table 4:** Screening of date.

Specialty	Adequate Theoretical Knowledge	Adequate Clinical Decision
	%	%
Intensivist, n = 129	78	66
Pulmonologist, n = 2	100	100
Cardiologist, n = 12	92	92
Cardiac surgeon, n = 5	100	100
Thoracic surgeon, n = 7	86	100
Anesthetist, n = 14	93	86
Internist, n = 53	74	60
General surgeon, n = 14	64	50

## Data Availability

The data that support the findings of this study are available from the corresponding author, A.M.A., upon reasonable request.
